# Research Refines Alcoholism Treatment Options

**Published:** 2000

**Keywords:** treatment research, screening and diagnostic method for AOD (alcohol or other drug) use, brief intervention, patient-treatment matching, twelve-step model, treatment outcome in Health Services Research (HSR), drug therapy, psychotherapy, interview, questionnaire

## Abstract

Every day, more than 700,000 people in the United States receive treatment for alcoholism. In recent years, much progress has been made in understanding how both psychological approaches and medications can help these patients achieve sobriety, including evaluation of existing treatment approaches and development of new ones. Continued research to refine therapies for alcoholism will have widespread benefits for alcohol-dependent people, for their families, and for society as a whole, which bears the weight of the enormous economic and social costs of problem drinking. This article reviews the current state of alcoholism treatment research.

For patients who are at-risk or problem drinkers but not alcohol dependent, health care providers can significantly reduce alcohol use and related problems by providing brief interventions, which consist of feedback and advice from the health care provider and agreement by the patient on a course of action. For patients who are alcohol dependent, numerous inpatient and outpatient treatment options are available. In recent years, escalating health care costs have propelled a shift from inpatient to outpatient treatment at all stages of recovery, although inpatient care remains more appropriate for patients with serious concurring medical or psychiatric conditions or in social environments that are not supportive of recovery. Whether inpatient or outpatient, the treatment can involve psychological approaches, medications, or a combination of the two.

Continued research to refine therapies for alcoholism will have widespread benefits for alcohol-dependent people, for their families, and for society as a whole, which bears the weight of the enormous economic and social costs of problem drinking. This article reviews the current state of alcoholism treatment research. It first discusses screening approaches and brief interventions that have been tested and validated in clinical settings. The article then summarizes research evaluating the effectiveness of the many psychological therapies currently used to treat alcoholism. Finally, the article describes recent advances in the development of medications both to treat alcohol dependence and to treat patients who suffer not only from alcohol dependence but also from psychiatric disorders, primarily depression.

## Screening and Brief Intervention for Alcohol Problems

One in 5 men and 1 in 10 women who visit their primary care providers meet the criteria for at-risk drinking, problem drinking, or alcohol dependence (Manwell et al. 1998). Furthermore, estimates suggest that alcohol dependence is found in 25 percent of persons seen in primary care settings who drink above recommended limits of alcohol use.^1^ Many of these patients do not consult alcohol treatment specialists; consequently, their primary health care providers have an important opportunity to identify and treat both potential and existing drinking problems.

Screening for alcohol-related problems usually involves asking the patient questions about drinking through structured interviews or self-report questionnaires; it may also involve laboratory tests to detect abnormalities associated with excessive alcohol consumption. Once a drinking problem—or a level of increased risk—has been identified, health care providers can take steps to help the patient minimize or prevent future problems. Often this intervention takes the form of advice or counseling to encourage the patient to alter behaviors that are contributing to the problem. In some cases, more detailed assessments are needed to specify the nature and extent of the problems so that appropriate treatment can be initiated.

### Screening for Alcohol Problems

Several alcohol screening instruments have been tested and validated in clinical settings, including brief, structured interviews that contain questions on the quantity and frequency of drinking; questionnaires that can be self-administered or used in an interview by a health professional; and clinical laboratory tests. These instruments should have high sensitivity and specificity.^2^

#### Interviews: Quantity-Frequency Questions

Currently, the standard of practice for most clinicians is to ask patients how much and how often they drink. To make the responses to these “quantity-frequency” questions uniform, a standard drink is defined as 12 grams of pure alcohol, which is equivalent to one 12-ounce beer or wine cooler, one 5-ounce glass of wine, or 1.5 ounces of 80-proof distilled spirits. The level of alcohol consumption that poses a risk for developing alcohol-related problems differs for men and women (National Institute on Alcohol Abuse and Alcoholism [NIAAA] 1995). Whereas men may be at risk if they have more than 14 drinks per week or more than 4 drinks on one occasion, women’s risk is increased with more than 7 drinks per week or more than 3 drinks per occasion (NIAAA 1995).

Quantity and frequency questions allow the clinician to estimate a patient’s risk directly. They are also easy to score and can be included as part of an office visit with minimum cost and effort. Examples of quantity and frequency questions include the following:

- On average, how many days per week do you drink alcohol?
- On a typical day when you drink, how many drinks do you have?
- What is the maximum number of drinks you had on any given occasion during the last month?

Such questions generally have high sensitivity in detecting persons who drink above recommended limits. However, some patients may understate their drinking, especially if they are alcohol dependent or are intoxicated at the time of the interview.

#### Questionnaires

The limitations of quantity and frequency questions led to the development of screening questionnaires designed for use in primary care settings. Most of these questionnaires focus on the consequences of patients’ drinking and their perceptions of their drinking behavior. Commonly used questionnaires whose effectiveness has been examined include the following:

- The CAGE instrument, which consists of four questions about the patient’s drinking and family or friends’ reactions to it; one or more “yes” answers indicate an increased risk of alcohol-related problems in both men and women.
- The Alcohol Use Disorders Identification Test (AUDIT), which was developed from a World Health Organization (WHO) collaborative project. It consists of 10 questions regarding the patients’ alcohol consumption, drinking behavior, and alcohol-related problems over the past year. The AUDIT’s sensitivity varies, however, depending on the study population and the cutoff score used. Furthermore, the AUDIT may be less effective for detecting alcohol problems among people who barely meet the criteria for at-risk drinking. Finally, the length of the AUDIT may make its administration cumbersome for some physicians or patients. It may be more useful for assessing patients after a possible problem has been detected by other methods.
- The Health Screening Survey and the Health Screening Questionnaire, which include questions about alcohol use as well as other health questions (e.g., on smoking, weight, exercise, and depression). Both instruments have adequate sensitivity and specificity.
- The Primary Care Evaluation of Mental Disorders (PRIME-MD), a relatively new instrument that includes the four CAGE questions and two questions on alcohol consumption. The PRIME-MD also can be used through telephone-assisted computer administration.
- The Trauma Scale, which consists of five-questions concerning fractures or dislocations, involvement in motor vehicle crashes, head injury, and injuries sustained in assaults, fights, or after drinking. The instrument is more sensitive than laboratory tests in detecting genuine cases of problem drinking and is also specific in ruling out “social,” nonproblem drinkers.
- The T-ACE and TWEAK questionnaires, which were developed specifically to screen for alcohol problems in pregnant women. Both tests are more sensitive than the CAGE questionnaire in identifying women who are drinking above recommended limits.

#### Laboratory Tests

Physicians also can uncover patients’ drinking problems through the use of biological analyses, such as blood and breath tests. Obtaining blood alcohol concentrations is particularly important in emergency departments, trauma centers, and other acute care settings for confirming patient self-reports and for managing patients who are to undergo surgery. For screening purposes in primary care settings, however, laboratory tests are not adequately sensitive or specific because they identify only about 10 to 30 percent of problem drinkers.

#### Assessment Instruments

For persons who screen positive through a questionnaire, interview, or laboratory test, clinicians can use several psychological assessment methods to determine the extent of the problems and develop a treatment plan. Several pencil-and-paper questionnaires are available to assess alcohol-related problems and physical dependence. Two examples are the Short Michigan Alcoholism Screening Test (S-MAST), a 13-question instrument widely tested in clinical settings, and the Short Alcohol Dependence Data Questionnaire (SADD), a 15-item assessment of dependence severity that has been widely used in alcoholism treatment studies. In addition, patients with alcohol problems should be assessed for mental health disorders, because the prevalence of depression, anxiety disorders, and other mental health problems is high among people with alcohol dependence, especially women.

#### Brief Intervention

Brief interventions are time-limited counseling strategies that are especially useful in busy, high-volume health care practices, where physicians are often pressed for time and have multiple priorities. These techniques can be used to reduce alcohol use in patients who drink above the recommended levels but who are not alcohol dependent. They may also be helpful in motivating patients with alcohol dependence to seek specialized alcohol treatment. In a brief intervention, the health care provider basically follows three steps:

- Stating the medical concern.
- Advising the patient to abstain from alcohol use (if alcohol dependent) or to cut down (if not).
- Agreeing on a plan of action.

Health care providers who employ brief interventions can also suggest techniques to help patients modify their behavior and suggest self-help material for the patients to read.

Brief interventions are a valuable resource for reducing patients’ problems with alcohol. Researchers have studied brief interventions in hospitals, in primary care clinics, on college campuses, in clinical research settings, and in urgent care settings. One study (Bien et al. 1993) analyzed 32 trials of brief interventions and found that most of these efforts had positive results, reducing alcohol use by up to 30 percent.

Three large, randomized, controlled clinical trials support the use of brief interventions in the family medicine setting, as follows:

- In a study of 909 patients conducted in the United Kingdom, the intervention group had significantly reduced drinking levels after 1 year compared with the control group and showed improved health (Wallace et al. 1988).
- In a U.S. study involving 720 patients—Project TrEAT (Trial for Early Alcohol Treatment)—problem drinkers receiving a brief intervention showed a greater reduction in their alcohol use at 12 months than did the control group (Fleming et al. 1997). Furthermore, binge drinking within the previous 30 days and excessive drinking within the previous 7 days was substantially reduced in the intervention group.
- A trial examining the effect of brief counseling interventions, delivered to 482 high-risk drinkers as part of routine primary care by physicians and nurse practitioners, found a significantly larger reduction in alcohol consumption in the intervention group compared with the control group receiving usual care (Ockene et al. 1999).

The efficacy of brief intervention in emergency care settings, such as hospital emergency departments and trauma centers, is a relatively new area of research. One recent study examined the effects of brief interventions in patients who had been admitted to a trauma unit for treatment of injuries and who had screened positive for alcohol problems (Gentilello et al. 1999). Among the patients for whom the intervention was completed, alcohol consumption was decreased significantly at 12 months compared with the control group. The difference was most pronounced in patients with mild-to-moderate drinking problems, whereas no benefit was seen in patients with severe drinking problems. Most importantly, at 12 months, the members of the intervention group had continued to decrease their alcohol intake, whereas control group members had returned to the level at which they had been drinking at the start of the study.

Another study evaluated the use of a brief motivational intervention to reduce alcohol use and alcohol-related consequences among adolescents treated in an emergency room following an alcohol-related event (Barnett et al. in press; Monti et al. 1999). In that study, both the intervention and the standard care groups had reduced their levels of consumption at 6 months, but the patients who received the brief intervention also had significantly lower rates of other alcohol-related problems (e.g., drinking and driving, traffic violations, and alcohol-related injuries) than did patients who received standard care.

Researchers have not yet determined the optimal length of an intervention and the optimal number of contacts with the patient. One international study, conducted by the WHO Brief Intervention Study Group, found no difference between a group receiving “simple advice” and a second group receiving “brief counseling” with more extensive intervention (WHO 1996). In contrast, results of a Canadian study suggest that multiple counseling sessions have a stronger treatment effect than a single visit for brief advice (Israel et al. 1996).

Several U.S. trials have tested the efficacy of brief interventions in special populations as follows:

- Two studies of college students have found that brief interventions can reduce alcohol use and alcohol-related problems over the long term (Marlatt et al. 1995, 1998).
- In the first brief intervention trial for pregnant women (Chang et al. 1999), both the intervention group and the control groups significantly reduced their alcohol use, and the difference between the two groups was minimal. It is possible that the intervention had no significant effect in this study because an assessment conducted at the outset of the study already accomplished the intended effect.^3^
- In a trial that included 175 Mexican-Americans who screened positive for alcohol abuse or dependence, all groups demonstrated significant improvement over time, with little difference between the intervention and control groups (Burge et al. 1997). Again, this may have been because the assessment procedure itself served as a brief intervention for the control group.
- In Project GOAL (Guiding Older Adult Lifestyles)—the first clinical trial to use a brief intervention with older adults who were problem drinkers—patients who received a brief intervention showed significant reductions in alcohol use in the previous week, episodes of binge drinking, and frequency of excessive drinking at 3, 6, and 12 months after the intervention (Fleming et al. 1999). This study provides the first direct evidence that brief physician advice can decrease alcohol use by older adults in community-based primary care practices.

### Areas for Future Research

The preponderance of evidence indicates that brief interventions delivered in primary care settings can decrease alcohol use for at least 1 year in persons who drink above recommended limits. Nevertheless, more research is needed to increase understanding of important related issues. For example, researchers must identify the essential components of a brief intervention in terms of its content, length, number of sessions, and the role of the health professional delivering it. Studies are also needed on whether brief interventions have a role in treating alcohol-dependent patients and whether they should be used routinely outside of primary care settings (e.g., in hospital emergency departments and trauma centers). Finally, researchers do not know whether brief interventions reduce morbidity, mortality, use of health services, and costs to the community as a whole.

Researchers also must identify ways to improve physicians’ use of brief interventions. Some evidence indicates that routine educational approaches may not be effective. In a systematic review of continuing medical education strategies, programs using peer discussion and sessions for practicing skills were more effective than formal courses with lectures and handouts, which had limited effect (Davis et al. 1995). Health care organizations also might consider peer review feedback, such as confidential performance reviews based on audits of medical records or written feedback by quality assurance committees, as one way of improving physician performance.

## Treatment of Alcohol Dependence With Psychological Approaches

A broad range of psychological therapies and philosophies are currently used to treat alcoholism, including social skills training, motivational enhancement, behavior contracting, cognitive therapy, marital and family therapy, aversion therapy, and relaxation training. These varied approaches have different levels of scientific support for their effectiveness. The task for the scientific community is to evaluate the various approaches and determine which strategies offer the best chances of successful outcome, with the understanding that some types of treatment may have better results for certain types of clients. Recent studies have primarily evaluated four aspects of psychological therapies. These include client-treatment matching, or the use of a client’s individual characteristics to select an appropriate treatment therapy; the effectiveness of professional treatments modeled on the 12 steps of Alcoholics Anonymous (AA); the value of supportive ancillary counseling for life problems that often co-occur with alcoholism; and the effects of variations in treatment intensity on outcomes.

### Client-Treatment Matching

No single psychological treatment approach has been found superior in promoting long-term recovery from alcoholism. Instead, many different treatment approaches appear to be equally effective. However, an overall similarity of outcomes may hide certain relationships whereby one type of treatment might produce better results for certain patients. For example, patients with long-term, stable marriages might be expected to benefit more from marital and family counseling approaches than would patients in shorter term or unstable relationships. Researchers have hypothesized that if they could identify important client characteristics and the treatments that work best for them, clients could be “matched” to the treatment from which they would benefit most.

A project called Matching Alcohol Treatments To Client Heterogeneity (Project MATCH) has provided the most careful and extensive test to date of the contributions of client-treatment matching to treatment outcome. In this multisite clinical trial, 1,726 clients were assigned randomly to a cognitive-behavioral, motivational enhancement, or 12-step facilitation treatment. The study’s primary goal was to evaluate whether treatments that were appropriately matched to the client’s needs produced better outcomes than did treatments that were not matched. The study investigated many client characteristics, among them gender, alcohol involvement, cognitive impairment, spirituality, motivation, social network support for drinking, alcohol dependence, level of anger, interpersonal dependency, prior AA involvement, self-efficacy, social functioning, antisocial personality disorder, type and severity of psychiatric disorder, religiosity, alcoholism type, and readiness to change. Each of the characteristics was evaluated to see whether clients who had different variations of the characteristic benefited differently from the various treatments provided.

Participants in the study were divided into two general treatment groups: one group received only outpatient treatment, and the other group received a more intense course of inpatient treatment followed by outpatient aftercare (hereafter labeled the after-care group). Outcomes were measured at 1 year following treatment for both groups and after 3 years for the outpatient group.

The results of Project MATCH yielded minimum support for matching the patient characteristics studied to the treatment types (Project MATCH Research Group 1998). Only 4 of the 21 potential matching characteristics resulted in different responses depending on the treatment received; these were severity of alcohol dependence in the aftercare group, psychopathology and anger in the outpatient group, and social network support for abstinence in both groups. These findings challenge the notion that patient-treatment matching is a prerequisite for optimal alcoholism treatment. The paucity of matching findings might be seen in the context of the finding that the three treatments studied were approximately equal in their efficacy. Any one of the treatments, therefore, would be expected to achieve results similar to the others.

### Professional Treatment Modeled on the 12 Steps of AA

Participation in AA or professional treatment programs based on the 12 steps of AA is the dominant approach to alcoholism treatment in the United States. Higher levels of AA attendance during and following professional treatment are consistently associated with better outcomes, but AA affiliation without professional treatment has not routinely resulted in improvement. In the Project MATCH trial just discussed, facilitation of participation in 12-step therapy (which was based on AA principles and encouraged AA participation) achieved outcomes at least as pronounced and durable, and by some measures even better, than other therapies (Project MATCH 1998). Another recent study, which evaluated 15 treatment programs offered through the U.S. Department of Veterans Affairs, indicated that patients in 12-step programs were more likely to become abstinent than were patients from cognitive-behavioral or mixed programs (Ouimette et al. 1997). Most of the other variables studied, however, such as mean alcohol consumption, alcohol dependence symptoms, use of other drugs, depression, anxiety, and arrests, revealed no significant differences between patients treated by different approaches.

How 12-step approaches function to produce positive treatment outcomes is another important research topic. One study found that five patient characteristics were related to both stronger affiliation with AA and better treatment outcome (Morganstern et al. 1997). The five characteristics were self-efficacy, commitment to abstinence, cognitive coping, behavioral coping, and primary appraisal of harm due to drinking. These results suggest that these five characteristics should be considered in future studies that seek to define the underlying mechanisms of effectiveness for 12-step-based treatments.

### Supportive Ancillary Services

Typically, clients entering treatment arrive with several other problems in addition to alcoholism, such as other drug abuse, mental health disorders (particularly depression), unemployment, domestic violence, and legal problems. Consequently, measures of treatment success often must consider a wide number of outcomes where improvement is sought (see sidebar). Furthermore, treatment for the alcoholism itself may have a greater chance of success if the other problems are being successfully addressed by appropriate services. For example, in one study, clients received either standard alcoholism counseling or standard alcoholism counseling plus adjunctive professional treatment sessions in areas that may result from, or contribute to, alcohol abuse (McLellan et al. 1997). Although patients in both groups had similar rates of abstinence after 6 months, those receiving adjunctive counseling were more likely to be working 20 or more hours per week and less likely to have family conflicts or to have been readmitted for alcohol or other drug (AOD) abuse treatment, arrested, or charged with a crime. In addition, clients assigned to adjunctive counseling stayed in treatment longer and were more likely to complete treatment.

**Psychological Treatment Outcomes: A Broad Perspective**

How effective are psychological interventions for persons experiencing alcohol abuse and alcoholism? At first glance, this question appears to be relatively straightforward. Nevertheless, attempts to provide simple answers to this question may overlook a number of important considerations. Experts in alcohol research urge other researchers, clinicians, and health care professionals to dismiss global statements about the effectiveness of alcoholism treatments and, instead, adopt a broader, more complex perspective on the outcomes of psychological interventions. Among the factors to consider are the following: patient diversity, context for treatment, outcomes other than changes in drinking behavior, and changes in outcomes over time.

***Patient Diversity***

Persons who receive treatment for alcohol abuse and alcoholism are a remarkably diverse group. The nature and severity of alcohol problems vary considerably, from serious forms of alcohol dependence to occasional problems with drinking. Consequently, judgments about outcomes must take individual patient characteristics into account.

***Context for Treatment***

Alcoholism treatment itself is a complex phenomenon. Specific psychological interventions are part of a larger context that includes expectancies of clinicians and clients as well as different settings, therapist characteristics, treatment intensity, treatment goals, and methods of payment. Thus, treatment actually represents a mix of these factors and attributes.

***Outcomes Other Than Changes in Drinking Behavior***

Treatment outcome is a multidimensional event. The usual standard for judging the effectiveness of alcoholism treatments is change in drinking behavior. Nevertheless, other equally important outcomes also deserve consideration. For example, it is important to understand how alcoholism treatment affects patients’ rates of illness and death, the nature of psychological disorders that accompany alcohol problems, and the use and costs of medical services triggered by alcohol misuse.

***Changes in Outcomes Over Time***

Relatively few patients remain in the same outcome status over a span of years. At any given time, many factors other than the treatment itself can contribute to positive or less-than-positive results. For example, the extent to which a patient’s social environment supports the changes resulting from treatment has an enormous effect on long-term outcomes. Thus, it is critical to consider the timing of evaluations of patient outcomes, to distinguish between short-term and long-term treatment effectiveness, and to examine the factors that hinder or support treatment effectiveness at different stages.

### Intensity of Services

Managed care has brought pressure to reduce treatment costs and eliminate unnecessary services. This makes the task of determining the optimal intensity (or duration and amount) of alcoholism treatment services more urgent. Few studies have compared the relative effectiveness of more versus less intensive forms of outpatient treatment. Emerging findings suggest that although treatment intensity may not predict long-term outcomes, it may affect the speed at which a person achieves some control over his or her drinking during treatment. Findings from the Project MATCH trial suggest that for outpatients, lower intensity treatment is slower than higher intensity treatment at helping patients achieve control over their drinking. Additional research is necessary on treatment intensity from a cost-effectiveness perspective (see also the article “Economic Analysis Aids Alcohol Research” in this issue).

## Treatment of Alcohol Dependence With Medications

In recent years, the development of new medications to treat alcohol dependence has initiated a new era in alcoholism treatment. Until 1995, the only medical treatment approved for use in the United States—the agent disulfiram—simply provoked intense physical symptoms, such as vomiting, upon the ingestion of alcohol. Over the past decade, however, advances in knowledge of the biology underlying drinking behavior have laid the groundwork for designing more targeted medications. For example, it is now known that multiple chemical messenger systems in the brain, called neurotransmitter systems, are involved in problem drinking. Several agents that affect different neurotransmitter systems have been tested in humans. In particular, one area of research has focused on a class of medications called opiate antagonists, which interfere with the activities of certain neurotransmitters that produce pleasurable effects (e.g., feelings of euphoria) after AOD consumption. In addition, researchers are evaluating medications that target different neurotransmitter systems involved in maintaining alcohol dependence that may contribute to drinking following a brief period of abstinence. Finally, pharmacology research has focused also on medications for coexisting conditions that can threaten recovery, particularly depression.

Although medications hold great promise, they cannot at present replace psychological treatments for people with alcohol dependence. These two classes of treatment strategies are complementary rather than competitive, in that pharmacologic agents may be combined effectively with skilled counseling to improve treatment outcomes.

### Medications for Alcohol Dependence

#### Opiate Antagonists

The treatment of alcohol dependence has benefited from an improved understanding of the mechanics of addiction and the ways in which certain medications can counter the effects of addictive drugs. Drugs like heroin and morphine, called opiates, act like chemicals the brain produces naturally, called endogenous opioids, which stimulate pleasurable feelings and suppress pain. Opiate antagonists bind to the brain’s receptors^4^ for endogenous opioids, thus blocking the desired effects of heroin and similar drugs while having no effect themselves. Although alcohol is not an opiate-like substance, opiate antagonists can also affect drinking, probably by blocking some of alcohol’s rewarding effects. Alcohol researchers have investigated the effects of two opiate antagonists, naltrexone and nalmefene. Naltrexone (ReVia) was approved by the U.S. Food and Drug Administration (FDA) for treating alcohol dependence in 1995, while nalmefene is still undergoing testing for approval.

Studies of naltrexone in humans have supported the hypothesis that opiate antagonists reduce the pleasurable effects associated with alcohol’s stimulation of the endogenous opioid system and related reward systems. For example, patients given naltrexone experienced less euphoria after drinking than did patients taking a placebo (Volpicelli et al. 1995). Thus, by reducing the positive reinforcement of drinking and increasing unpleasant effects, naltrexone may help people who have relapsed to refrain from heavy drinking. Other findings suggest that naltrexone blocks both the chemical changes in the brain elicited by environmental cues before drinking and the “priming” effects of alcohol during drinking—both of which are associated with the urge to drink and loss of control over drinking (O’Malley et al. 1995). Finally, researchers found that patients given naltrexone drank less frequently, and when they drank, they consumed less alcohol, regardless of their demographic traits and the behavioral treatments they received.

Several investigators have examined the side effects of naltrexone. In general, no serious adverse events are associated with naltrexone treatment at the typical dose (i.e., 50 milligram per day). At much higher doses, however, naltrexone is associated with adverse liver effects. As a result, this medication is not appropriate for patients with acute hepatitis or liver failure, both of which can occur in long-term alcoholic patients.

Follow-up studies of patients who have used a medication can yield important information about its long-term effects as well as the potential for relapse when the medication is discontinued. Researchers found that naltrexone-treated patients were less likely to relapse during the first month after they stopped using the medication, to drink heavily during the first 4 months after treatment, and to meet the criteria for alcohol abuse and dependence 6 months after treatment than were patients receiving a placebo (O’Malley et al. 1996). These findings provide support for naltrexone’s long-term efficacy, at least in some patients.

Researchers also obtained the following results regarding the optimal use of naltrexone:

- For patients who have difficulty achieving abstinence during initial treatment, use of naltrexone beyond the standard 12-week treatment period may be helpful.
- Patients who anticipate periods of relapse risk, such as an upcoming vacation, or experience sudden stressful events, such as the death of a friend, may benefit from using naltrexone again for short periods until they feel more secure about avoiding relapse.
- Patients who may derive the greatest benefit from naltrexone may be those who experience an intense urge to drink and who have physical symptoms, such as chronic pain or discomfort, coupled with poor cognitive functioning, such as impaired learning skills and memory.

Nalmefene, not yet approved by the FDA for treatment of alcoholism, is structurally similar to naltrexone. It has been shown to be effective in reducing relapse to heavy drinking, and it does not produce liver toxicity at high doses. Replicating the results of naltrexone studies with this structurally similar compound supports the importance of further research on opiate antagonists to treat alcohol dependence. Nalmefene may be an option for patients who experience adverse side effects from naltrexone or who do not respond to that drug.

#### Acamprosate

The medication acamprosate interacts with different biochemical pathways in the brain than those affected by opioid antagonists. Although the precise mechanism of action is still under investigation, acamprosate is known to affect two neurotransmitter systems involved in maintaining alcohol dependence: the glutamate system and the gamma-aminobutyric acid (GABA) system. Chronic alcohol exposure disrupts both systems, causing changes that may persist for many months following withdrawal. Acamprosate may act by restoring normal activity in these systems.

Acamprosate has been used to treat more than 1 million alcohol-dependent people in more than 30 countries. In the United States, acamprosate is currently being tested in clinical trials. One study evaluating the safety and efficacy of acamprosate across 21 different treatment settings has recently been completed, and the data are now being analyzed.

In 11 clinical trials in Europe, researchers compared the effectiveness of acamprosate with that of a placebo. In 10 of the studies, patients on acamprosate experienced higher abstinence rates, and those who did resume drinking, experienced a significantly longer period of abstinence until their first drink than did patients on the placebo. Furthermore, when researchers analyzed pooled data from all 11 trials, the patients on acamprosate had significantly higher rates of abstinence and treatment attendance than those on the placebo, as well as longer alcohol-free periods. The effects of acamprosate were evident during the first 30 to 90 days of treatment, the interval during which the risk of drinking is the highest and pharmacologic support may be most effectively implemented.

No single study has directly compared acamprosate with naltrexone. However, researchers have compared each medication with a placebo in separate studies that yielded quite similar results. Because several neurotransmitter systems are involved in maintaining alcohol dependence, the effect of any single medication on alcohol intake may be modest. Both acamprosate and naltrexone are well tolerated by patients, and the medications’ actions on different neurotransmitter receptors may lead to different effects on drinking outcomes (such as preventing relapse to heavy drinking or prolonging abstinence). Thus, NIAAA is currently funding a cooperative, multicenter study that will test acamprosate and naltrexone, both alone and in combination, and evaluate their use compared with a placebo in conjunction with behavioral interventions of either moderate or minimum intensity.

#### Serotonergic Agents

The neurotransmitter serotonin affects multiple actions in the brain, including the regulation of mood states, appetite, and sleep. The exact nature of the relationship between serotonin and alcoholism is unknown, but various theories exist. Several of these theories are based on the premise that people with alcohol dependence have lower than normal levels of serotonin in the brain. Consequently, researchers have examined whether alcohol intake could be reduced by medications that increase the amount of serotonin available for binding to receptors on nerve cells in the brain. Among the “serotonergic” agents that have been evaluated for alcoholism treatment are sertraline (Zoloft), fluoxetine (Prozac), and several other “selective serotonin reuptake inhibitors” (SSRIs), a class of drugs developed in the 1980s to treat depressive disorders. Thus far, however, studies on the effectiveness of serotonergic agents in reducing alcohol intake have shown only a mild and transient effect in moderate drinkers and no effect in alcohol-dependent patients (for a review of these studies, see Litten et al. 1996). Although serotonergic agents have not yet fulfilled the promise they once seemed to offer in treating alcoholism, they may be effective for treating psychiatric conditions that often co-occur with alcohol dependence, such as depression.

### Medications for Patients With Both Alcoholism and Depression

People with alcohol dependence often experience symptoms of depression when they stop drinking. For most individuals, these symptoms disappear or wane during the first 1 or 2 weeks of abstinence. Patients who continue to report serious depressed feelings after the first week of abstinence, however, are likely to have a depressive disorder that coexists with their alcohol dependence. These patients are often referred to as having “comorbid depression” or a “dual diagnosis.” If the depression is left untreated, many of these patients will relapse. Thus, accurate diagnosis and swift treatment of depression are critical in the care of alcohol-dependent patients.

Investigators have examined different types of antidepressant agents for dually diagnosed patients, including the older tricyclic antidepressants (e.g., imipramine and desipramine) that have been available since the 1960s, and the newer SSRIs, such as sertraline and fluoxetine. Regardless of the type of antidepressant used, depressed, alcohol-dependent patients who take antidepressants have better outcomes than do those who take a placebo. Some participants in these antidepressant trials, however, continued to drink even though their depression lifted, demonstrating the need for additional interventions specific to drinking.

In making decisions about diagnosis and treatment of depressed alcohol-dependent patients, some clinicians distinguish between primary depression, which occurs before the onset of alcoholism, and secondary depression, which occurs afterwards. Studies have shown that antidepressant medications can improve mood and reduce drinking regardless of whether the patients’ depression is primary or secondary.

## Summary

The U.S. health care system offers a great opportunity to identify and treat the majority of people who are adversely affected by alcohol use disorders. Numerous screening tests can help identify at-risk drinkers, and research suggests that brief advice and counseling can reduce their levels of drinking and health care utilization. The challenge, however, is to incorporate alcohol screening and brief intervention practices into existing clinical activities and prevention programs in these systems of care. Changing systems of health care is a complex endeavor, similar to changing patient alcohol use—education is a critical first step, but the next, and far more difficult step, is taking action.

Treatment outcome studies have repeatedly found large and sustained reductions in drinking among persons seeking help for alcoholism. Still, many individuals continue to suffer problems with alcohol following treatment. Researchers are trying to improve treatment by undertaking further investigations of the factors and conditions that might improve outcomes after both psychological treatment and medication therapies. Recent findings on psychological therapies have led to four conclusions. First, matching broad categories of client characteristics to treatment modality does not substantially improve overall treatment outcomes. Second, professional treatments based on 12-step approaches can be as effective as other therapeutic approaches and may actually achieve more sustained abstinence. Third, supportive ancillary services can be effective in remediating common problems that co-occur with alcoholism. Fourth, higher intensity outpatient treatment (i.e., 12 weekly sessions) may help a client gain control of drinking more quickly.

Currently, clinical trials are under way to search for new and more effective pharmaceutical agents to treat alcohol-dependent individuals. Additional studies will be needed to identify the most appropriate medications for different patient subgroups. To date, younger men have constituted the majority of subjects in studies of the pharmacotherapy of alcoholism. Future research will need to examine data from women, older adults, and other subgroups to determine the medications that are most effective and acceptable, with the fewest adverse side effects, for each group of patients.

## Footnotes

1 For men, recommended drinking levels are no more than two drinks per day or four per occasion; for women, those levels are no more than one drink per day or three per occasion.

2 Sensitivity is a measure of an instrument’s accuracy in detecting persons with the problem in question. A tool with high sensitivity only rarely gives a “false-negative” result for someone who is actually positive. Conversely, specificity is a measure of how well the tool excludes people who do not have the problem; a tool with high specificity only rarely gives “false-positive” results.

3 Other possible explanations for the lack of treatment effect are the fairly high rate of abstinence among the women at the time of random assignment, as well as the tendency of many women to reduce drinking during pregnancy.

4 Receptors are proteins on the surface of nerve cells that serve as “docking molecules” for neurotransmitters.
